# Violencia sexual en un municipio de Colombia: características de las víctimas y de sus victimarios, 2011-2020

**DOI:** 10.7705/biomedica.6460

**Published:** 2022-09-02

**Authors:** Camilo Noreña-Herrera, Sergio Andrés Rodríguez

**Affiliations:** 1 Facultad de Enfermería, Universidad CES, Medellín, Colombia Universidad CES Facultad de Enfermería Universidad CES Medellín Colombia; 2 Dirección de Gestión del Conocimiento y Planeación en Salud, Secretaría de Salud de Envigado, Envigado, Colombia Dirección de Gestión del Conocimiento y Planeación en Salud Secretaría de Salud de Envigado Envigado Colombia

**Keywords:** delitos sexuales, violencia doméstica, violencia de género, violencia contra la mujer, violación, acoso sexual, Sex offenses, domestic violence, gender-based violence, violence against women, rape, sexual harassment

## Abstract

**Introducción*.*:**

La violencia sexual es un problema de salud pública inscrito en las agendas sociales y políticas a nivel global. Representa una urgencia médica que se atiende en los servicios de salud, lo que los convierte en la principal fuente de su registro en los sistemas de vigilancia en salud pública.

**Objetivo.:**

Analizar las características sociodemográficas de las víctimas de violencia sexual y de sus victimarios en el municipio de Envigado, Antioquia, entre el 2011 y el 2020.

**Materiales y métodos.:**

Se hizo un estudio transversal descriptivo a partir de todos los registros de violencia sexual notificados en el Sistema de Vigilancia en Salud Pública de la violencia de género e intrafamiliar de Envigado (código de ficha INS-875), durante el periodo 2011-2020.

**Resultados.:**

Se registraron 807 casos de violencia sexual entre 2011 y 2020; el 62,0 % correspondió a casos de abuso sexual a personas menores de 18 años; el 82,3 % de las víctimas estaba constituido por mujeres adolescentes agredidas por familiares; los principales agresores fueron hombres (99,1 %), con una mediana de edad 26 años.

**Conclusión.:**

La violencia sexual es un problema en aumento; en el 2020, año del confinamiento poblacional por la Covid-19, las tasas en mujeres y durante la primera infancia, la adolescencia y la juventud, fueron las más altas del periodo de estudio.

## Abstract (en)

**Introduction:**

Sexual violence is a public health problem included in political and social agendas worldwide. It is a medical emergency seen in health services, which are the main source of record-keeping by means of public health surveillance systems.

**Objective:**

To analyze the sociodemographic characteristics of victims of sex offenses and their offenders in the municipality of Envigado, Antioquia, between 2011 and 2020.

**Materials and methods:**

This was a descriptive cross-sectional study that analyzed all the sexual violence records reported in the Envigado Public Health Surveillance System for gender and intrafamilial violence (file code INS-875) during the 2011-2020 period.

**Results:**

A total of 807 cases of sexual violence were recorded between 2011 and 2020; 62.0% were cases of sexual abuse against persons under 18 years of age, and 82.3% of the victims were adolescent women suffering sexual violence exerted by relatives. The main aggressors were men (99.1%) with a median age of 26 years.

**Conclusion:**

Sexual violence has increased over time and in 2020, the year of population confinement due to COVID-19, the rates in women and in the life courses of early childhood, adolescence, and youth were the highest during the study period.

La violencia sexual es un problema de salud pública incluido en las agendas sociales y gubernamentales de múltiples entidades de carácter global ^1^^-^^5^. Afecta la dignidad, es una afrenta a los derechos humanos de las personas que la padecen ^6^^-^^9^, y está anclada a condiciones de inequidad social que se expresan en las desigualdades de género, en relaciones de poder fincadas en las condiciones del ingreso económico, la edad e, incluso, el poder físico y armado ^10^^,^^11^, así como en aspectos culturales como las representaciones sociales sobre la propiedad de los cuerpos de las personas, los usos y las costumbres ^11^^-^^13^. Se desprende de una deficiente enunciación y un limitado desarrollo programático en torno a los derechos sexuales y reproductivos en las agendas públicas, debido a presiones políticas y religiosas que generan tabúes sobre la sexualidad y afectan la disponibilidad y el acceso de las víctimas a servicios de salud con profesionales especializados, y a la protección y la justicia debidas ^14^.

La definición de la Organización Mundial de la Salud (OMS) plantea que la violencia sexual no solamente se circunscribe a los actos abusivos contra el cuerpo de las personas, sino que también incluye los acosos y las circunstancias en las que median relaciones de poder que socavan su cuerpo, su sexualidad y su dignidad en cualquier ámbito, incluida la familia y el entorno cercano ^10^.

La evidencia científica y los reportes mundiales develan que la violencia sexual afecta a un importante número de personas, sin distinciones de sexo, edad, condición socioeconómica, racial o cultural, pero sí con diferencias en cuanto a su prevalencia por grupos según las condiciones sociales y demográficas señaladas, pues la mayoría de las víctimas son mujeres, niños y adolescentes (^11^^,^^12^^,^^15^^-^^18^, situación que se acrecent*ó* durante el confinamiento poblacional por la pandemia de Covid-19 ^19^^-^^21^.

Debe considerarse que, entre los hombres, la forma de agresión usual se concreta en lesiones contra su integridad física y en el homicidio, en tanto que los actos violentos contra las mujeres son principalmente de carácter sexual, lo que incluye las violaciones sexuales sistemáticas en el contexto de la esclavitud sexual, los embarazos y la esterilización forzada, el aborto y la utilización coercitiva y forzada de anticonceptivos ^22^. La evidencia científica indica que las niñas y mujeres frecuentemente sufren violencia sexual a manos de personas que no son su pareja; en Latinoamérica y el Caribe, las subcategorías comunes de esta modalidad son la violación, el abuso sexual de niñas y jóvenes, la trata y la explotación sexual, la violencia sexual durante el proceso de migración, el acoso sexual en el lugar de trabajo y la violencia sexual en situaciones de emergencia y conflicto o posconflicto ^17^.

La violencia sexual puede provenir de personas de la misma familia, de la pareja sentimental y de integrantes de la comunidad conocidos o extraños, y se interrelaciona con dimensiones sociales, políticas y económicas de las víctimas y los contextos. Murad, *et al.*, han desarrollado modelos explicativos de la violencia contra las mujeres con cuatro “énfasis comprensivos”:

“[…] el primero se fundamenta en los aspectos personales de la víctima y el victimario; otro en el que la violencia es un elemento que se transmite de generación en generación; un tercero donde (sic) la violencia es una forma más de socialización y, el último, percibe la violencia contra las mujeres como una expresión de las inequidades entre los géneros […]” ^23^.

La violencia sexual tiene implicaciones del orden individual, familiar, económico y social, que pueden ser graves y duraderas ^10^^,^^17^. Entre las consecuencias para la salud sexual y reproductiva, están las infecciones de transmisión sexual (ITS), incluido el VIH/sida, los embarazos no planeados ni deseados, la disfunción sexual y los problemas ginecológicos ^17^. Además, si se presenta durante el embarazo, aumenta la probabilidad de aborto espontáneo, muerte prenatal, parto prematuro y bajo peso al nacer del niño, y afecta la conexión biopsicosocial entre la madre y su hijo ^10^.

Entre los efectos en la salud física, se encuentran cefalea, lumbalgia, dolor abdominal, fibromialgia, trastornos gastrointestinales, limitaciones de la movilidad y deterioro del estado de salud. En algunos casos, se producen traumatismos mortales ^24^. En el ámbito de la salud mental es común que las mujeres víctimas de violencia infligida por su pareja experimenten angustia emocional y comportamientos suicidas ^10^^,^^25^, depresión, trastorno de estrés postraumático, insomnio y trastornos alimentarios ^24^^,^^25^.

La violencia sexual en la infancia puede generar estrés postraumático temprano o más tarde en la vida, trastornos de ansiedad e, incluso, intentos de suicidio. Además, en los casos de violencia intrafamiliar, se ha documentado el consumo de sustancias psicoactivas ^26^, además de trastornos alimentarios como la bulimia nerviosa ^27^.

Por otra parte, los costos sociales y económicos repercuten en toda la sociedad. Las mujeres, por ejemplo, se ven aisladas e incapacitadas para trabajar, pierden su sueldo, dejan de participar en las actividades cotidianas, y ven menguadas las fuerzas para cuidar de sí mismas y de sus hijos ^24^. En muchos casos, las consecuencias de la violencia sexual exigen la atención en instituciones de salud. Además, en la normatividad colombiana (Ley 1257 de 2008, Código Penal), esta se tipifica como un delito de tipo sexual, por lo que las víctimas deben acudir a entidades judiciales e investigativas para denunciar el caso, de manera que se pueda judicializar al agresor.

Por último, según el Instituto de Medicina Legal, en el 2019 se realizaron en Colombia 26.158 exámenes médicos legales por presuntos delitos sexuales, de los cuales el 73,7 % correspond*ía* a mujeres menores de 18 años (con mayor prevalencia entre los 12 y los 17 años) y, el 12,4 %, a mayores de edad (con mayor prevalencia entre los 18 y los 28 años). Se puede decir, entonces, que en nuestro país la prevalencia de la violencia sexual es mayor en las mujeres jóvenes. En el caso del departamento de Antioquia, se evidencia un comportamiento similar, pues se report*ó* que el 84,5 % de los exámenes se hizo en mujeres, principalmente en las menores de 18 años ^28^.

En este contexto, la ausencia de evidencia científica en torno a la violencia sexual en el municipio de Envigado ha impedido contar con los datos que respalden la formulación de estrategias integrales, intersectoriales y permanentes para garantizar el derecho a la salud de la población afectada. Según el Departamento Nacional de Estadísticas (DANE), la población total de Envigado en el 2020 era de 242.197 habitantes, 130.695 (54 %) de ellos mujeres y 111.502 (46 %) hombres, asentados predominantemente en la zona urbana (96,7 %), en tanto que, en la zona rural que es la de mayor extensión, solo vivía el 3,3 %. En cuanto a los grupos de edad, el 5,3 % (12.934) de los habitantes correspondía a la primera infancia (0 a 5 años), el 5,5 % (13.420) a la infancia (6 a 11 años), el 6,3 % (15.331) a la adolescencia (12 a 17 años), el 16,1 % (39.018) a la juventud (18 a 28 años), el 46,6 % (112.806) a la adultez y el 20,1 % (48.688) restante a adultos de 60 años o más ^3^.

Dada la importancia de ampliar el conocimiento sobre la violencia sexual y visibilizar su magnitud como problema de salud pública, en este estudio se analizaron las características sociodemográficas de las víctimas de violencia sexual y de sus victimarios entre el 2011 y el 2020 en el municipio de Envigado, a partir de los datos registrados en el Sistema de Vigilancia Epidemiológica, Sivigila, de la Secretaría de Salud municipal.

## Materiales y métodos

Este estudio epidemiológico transversal se inscribe en el marco del proyecto “Diagnóstico y medición de indicadores que ayuden al reconocimiento de las condiciones de salud, retos de atención y la generación de planes de acción en salud en el municipio de Envigado para hacer frente a las violencias sexuales”. La información provino del Sistema de Información para la Vigilancia en Salud Pública (Sivigila) de la Secretaría de Salud de Envigado, en tanto que las tasas poblacionales de violencia sexual se estimaron a partir de los datos del DANE.

Se incluyeron en el estudio todos los eventos de violencia sexual y violencia de género e intrafamiliar de Envigado notificados al Sivigila (código de ficha 875 del Instituto Nacional de Salud) durante el periodo 2011-2020, por las instituciones prestadoras de servicios de salud (IPS) del municipio, las cuales actúan como las unidades primarias generadoras de datos (UPGD). Los datos se recolectaron a partir de lo consignado en la ficha 875 por los profesionales de la salud de las IPS y otras UPGD, quienes registran estos eventos en el curso de la prestación de los servicios de salud considerando las variables definidas.

Para la homologación y control de calidad de los datos registrados entre el 2011 y el 2020, se tomaron las diez bases de datos del Sivigila que recogen lo concerniente a la vigilancia en salud pública de la violencia de género e intrafamiliar. Se consolidó una única base de datos después de analizar las fichas y sus instructivos de diligenciamiento, utilizando tablas de homologación para recodificar, transformar y crear nuevas variables. Se excluyeron aquellos registros cuya variable de “ajuste” arrojó los valores “6” y “D”, que corresponden a casos “descartados” y “descartados por error de digitación”, respectivamente.

La variable principal del estudio se determinó como la “naturaleza de la violencia sexual”; después de homologar entre los años, se la recodificó con base en la Ley 1257 de 2008, la Ley 1236 de 2008 y la Resolución 459 del 2012 del Ministerio de Salud, en las siguientes tres categorías:

1. abuso sexual, es decir, todos los actos sexuales contra niños, niñas y adolescentes entre los 0 y los 17 años, que incluyen el acceso carnal violento, la explotación sexual comercial (ESCNNA), y cualquier acto calificado como acoso en menores de 14 años;
2. acceso carnal violento, circunscrito a los actos sexuales violentos contra personas de 18 años o mayores, entre ellos, las violaciones, los asaltos sexuales y la explotación sexual comercial; y
3. acoso sexual, que integró los actos sexuales en personas de 14 años o más que no incluyen penetración, como los tocamientos, persecuciones, exposición a la pornografía y demás acosos físicos, verbales o mediados por la virtualidad con pretensiones sexuales.

La variable del estrato socioeconómico se analizó con base en lo establecido en la Ley 142 de 1994, “Por la cual se establece el régimen de los servicios públicos domiciliarios y se dictan otras disposiciones”, en cuyo artículo 102 se clasifican los estratos socioeconómicos de las viviendas con servicios públicos en seis categorías o grupos: 1) bajo-bajo, 2) bajo, 3) medio- bajo, 4) medio, 5) medio alto, y 6) alto. Ello permitió determinar, de alguna manera, el nivel de vulnerabilidad económica de la población.

El análisis de la información incluyó descripciones de persona, tiempo y lugar, mediante estadísticas de resumen y tendencia central para las variables con escala de medición continua, y de frecuencias relativas y absolutas para las variables nominales. Además, se calcularon tasas poblacionales específicas para cada año, considerando la población residente en Envigado. La información estadística se procesó en el programa Jamovi, versión 1.6.23.

Dada la existencia de otras posibles fuentes de información sobre los casos ocurridos en el municipio de Envigado, se solicitó información al Instituto Nacional de Medicina Legal y Ciencias Forenses, pero no fue posible obtener acceso a sus bases de datos para el cruce de los registros debido a la reserva de la información por parte de esta institución.

En concordancia con la Resolución 8430 de 1993 del Ministerio de Salud de Colombia, el proyecto fue considerado por el comité de ética de la investigación de la Universidad CES como un proyecto sin riesgo (Acta 177 de 2021) por utilizar exclusivamente fuentes de información secundarias.

## Resultados

### Características de los casos de violencia sexual notificados en el periodo de estudio

En el cuadro 1 se presentan las características de los casos de violencia sexual notificados al Sivigila en Envigado entre el 2011 y el 2020. Se registraron 807 casos de violencia sexual, con un promedio anual de 81 casos (desviación estándar, DE, ±46 casos). A partir del 2017, los casos anuales superaron los 120 casos y, entre el 2017 y el 2020, se acumula el 63,1 % del total.

**Cuadro 1:** Características de los casos de violencia sexual notificados al Sivigila en Envigado, 2011-2020

Variables		n	%
Año de notificación			
	2011	8	1,0
	2012	19	2,4
	2013	49	6,1
	2014	71	8,8
	2015	71	8,8
	2016	80	9,9
	2017	120	14,9
	2018	125	15,5
	2019	139	17,2
	2020	125	15,5
	Total	807	100
Tipo de violencia sexual			
	Abuso sexual	501	62,0
	Acceso carnal violento	274	34,0
	Acoso sexual	32	4,0
	Total	807	100
Municipio donde ocurrió el hecho			
	Envigado	506	62,7
	Medellín	157	19,5
	Itagüí	44	5,5
	Sabaneta	26	3,2
	Bello	20	2,5
	Otros municipios	54	6,7
Total		807	100

Fuente: datos del programa de Vigilancia Epidemiológica de la Secretaría de Salud de Envigado

En cuanto al tipo de violencia sexual, el mayor porcentaje (62,0 %) correspondió a los casos de abuso sexual (501 casos), el 34,0 %, a casos de acceso carnal violento en la población mayor de 18 años (274 casos), y el 4,0 %, a casos de acoso sexual (32 casos), es decir, persecuciones, hostigamientos o asedios físicos o verbales con fines sexuales en personas de 14 años o más no consentidos por ellas.

De los 807 casos de violencia sexual notificados y registrados en el Sivigila municipal, 506 (62,7 %) fueron contra personas residentes en el municipio y los otros 301 (37,3 %) contra no residentes en el municipio, pero que fueron atendidos en sus servicios de salud.

### Características sociodemográficas de la población de estudio

En el cuadro 2 se presentan las características sociodemográficas de la población de estudio. El 82,3 % (664) de los casos correspondió a mujeres, y de estos, siete fueron contra personas con algún tipo de discapacidad y edades entre los 8 y los 73 años, en tanto que, en cuatro casos, las víctimas tenían algún tipo de diagnóstico de carácter psiquiátrico. Además, el 80,3 % (533) de los casos se presentó contra mujeres en edad reproductiva (entre los 10 y los 49 años), de las cuales, el 5,8 % ^31^ estaba en gestación (entre la semana 5 y la 40) en el momento del evento.

**Cuadro 2:** Características sociodemográficas de las víctimas de violencia sexual en Envigado, 2011-2020

Variables		n	%
Hombre		143	17,7
Mujer		664	82,3
Total		807	100
Etapa de vida			
	Primera infancia (0 a 5 años)	175	21,7
	Infancia (6 a 11 años)	122	15,1
	Adolescencia (12 a 17 años)	209	25,9
	Juventud (18 a 28 años)	207	25,7
	Adultez (29 a 59 años)	84	10,4
	Vejez (60 años y más)	10	1,2
	Total	807	100
Etnia de la víctima			
	Indígena	3	0,4
	Rom (gitano)	3	0,4
	Afrodescendiente (R-P)	6	0,7
	Ninguna	795	98,5
	Total	807	100
Orientación sexual			
	Heterosexualidad	315	39,0
	Homosexualidad	66	8,2
	Bisexualidad	15	1,9
	Asexualidad	6	0,7
	Otra	97	12,0
	Sin diligenciar	308	38,2
	Total	807	100
Estrato socioeconómico de las víctimas			
	Uno	13	1,6
	Dos	48	5,9
	Tres	128	15,9
	Cuatro	26	3,2
	Cinco	10	1,2
	Seis	2	0,2
	Sin dato	580	71,9
	Total	807	100
Aseguramiento en salud			
	Contributivo	568	70,4
	Subsidiado	126	15,6
	Excepción	58	8,1
	No asegurado	47	5,8
	Sin dato	1	0,1
Total		807	100

Fuente: datos del programa de Vigilancia Epidemiológica de la Secretaría de Salud de Envigado

El promedio de edad de las víctimas fue de 16 años (DE=±13 años), en un rango entre el año y los 88 años de vida. La edad más frecuente correspondió a niños y niñas de cuatro años. Al agrupar la edad por el momento en el curso de vida, los casos se concentraron en la primera infancia (1 a 5 años), la infancia (6 a 11 años) y la adolescencia (12 a 17 años), con el 62,7 % (506) de los casos. De estos, siete casos correspondieron a niños y niñas bajo protección estatal en el Instituto Colombiano de Bienestar Familiar (cuatro en mujeres y tres en hombres), y el 25,7 % de los casos se presentó en la juventud (18 y 28 años), es decir que el 88,4 % de todos los casos de violencia sexual en Envigado se presentó en la población entre 0 y 28 años. Solo un 11,6 % de los casos se dio en la población mayor de 28 años: el 10,4 % en la adultez (29 a 59 años) y el 1,2 % (10 casos) en la vejez (población de 60 años o más).

En cuanto a la condición étnica, 12 (1,5 %) casos de violencia sexual se presentaron contra mujeres que se reconocían como pertenecientes a grupos étnicos, y se distribuyeron de la siguiente manera: tres casos en mujeres indígenas entre los 18 y los 40 años; tres casos en mujeres gitanas entre los 3 y los 19 años, y seis casos en mujeres de la población afrodescendiente (entre los 3 y los 32 años). En los otros 795 casos (98,5 %), no se reportó adscripción a ningún grupo étnico.

La orientación sexual más frecuente (39,0 %) en las víctimas correspondía a la heterosexualidad, el 8,2 % declaró preferencias hacia la homosexualidad (66 casos) y el 38,2 % de los reportes carecía de información referente a esta variable de interés (308 casos). Con relación a la nacionalidad, tres (0,4 %) casos se presentaron en personas procedentes de otro país (Venezuela) y, los demás (804 casos), en personas de nacionalidad colombiana.

Considerando el estrato socioeconómico, el 23,4 % de los casos se presentó en los estratos socioeconómicos de bajos ingresos (1, 2 y 3), siendo el estrato tres el de mayor porcentaje (15,9 %); cabe señalar que el 71,9 % de los registros no tenía información sobre el estrato socioeconómico. En cuanto al régimen de seguridad social en salud, el 5,8 % (47) de las víctimas no se encontraba afiliada, el 70,4 % (568) pertenecía al régimen contributivo y 126 (15,6 %) al régimen subsidiado.

Además, 45 casos afectaron a personas con antecedentes de violencia en sus vidas (conflicto armado, desplazamiento forzado, violencia sexual), 40 de ellos afectaron a mujeres entre los 3 y los 73 años, y cinco, a hombres entre los 2 y los 22 años. Asimismo, se presentó un caso de violencia sexual contra un hombre de 57 años en situación de calle. Por último, cabe señalar que no se reportaron casos entre la población privada de la libertad, excombatientes, o niños y niñas en hogares y jardines infantiles.

### Características sociodemográficas de los victimarios

En el 89,1 % (719 de los 807 casos reportados), el agresor fue un hombre, en el 5,5 % ^44^, fue una mujer, en el 0,7 % (6), las víctimas no pudieron determinar el sexo de la persona agresora, en tanto que, en el 4,7 % ^38^, no se registró esta información. Con respecto a la edad del agresor, en el 72,6 % (513) de los casos esta no fue reportada. En los 294 registros con información sobre la edad, la mediana fue de 26 años (RIC=17 años), siendo 80 años la edad máxima entre los agresores.

En cuanto al parentesco entre agresor y víctima, en el 58,6 % (473) de los casos existía algún tipo de parentesco familiar. En el cuadro 3 se evidencia que, de los casos con algún parentesco, la pareja (19,2 %) o expareja (9,9 %) fue la agresora, con el 29,1 % de los casos. El padre fue el agresor en el 11,2 % de los casos y, otros familiares, en el 48,0 %. Cabe señalar que la ficha de notificación no permite la identificación específica de los otros tipos de familiares. Además, en el cuadro se aprecia que, según el sexo y el curso de vida de las víctimas, un mayor porcentaje de hombres sufre violencia sexual por parte de sus padres, especialmente durante la primera infancia (24,2 %), y por parte de los tíos, durante la infancia (15,4 %). En la adolescencia y la juventud, el principal agresor(a) fue la pareja o expareja.

**Cuadro 3:** Distribución porcentual del tipo de parentesco familiar de los agresores según el sexo y la etapa de vida de las víctimas de violencia sexual, Envigado, 2011-2020

			Tipo de parentesco familiar								
Sexo	Etapa de vida		Pareja	Padre	Expareja	Tío	Primo	Padrastro	Madre	Otro familiar	Total
Hombres	Primera infancia	n	0	8	0	2	3	1	0	19	33
		%	0,0	24,2	0,0	6,1	9,1	3,0	0,0	57,6	100
	Infancia	n	0	2	0	4	0	0	1	19	26
		%	0,0	7,7	0,0	15,4	0,0	0,0	3,8	73,1	100
	Adolescencia	n	1	1	1	0	0	0	0	5	8
		%	12,5	12,5	12,5	0,0	0,0	0,0	0,0	62,5	100
	Juventud	n	1	0	1	0	0	0	0	5	7
		%	14,3	0,0	14,3	0,0	0,0	0,0	0,0	71,4	100
	Adultez	n	0	0	1	0	0	0	0	3	4
		%	0,0	0,0	25,0	0,0	0,0	0,0	0,0	75,0	100
	Vejez	n	0	0	0	0	0	0	0	1	1
		%	0,0	0,0	0,0	0,0	0,0	0,0	0,0	100,0	100
	Total	n	2	11	3	6	3	1	1	52	79
		%	2,5	13,9	3,8	7,6	3,8	1,3	1,3	65,8	100
Mujeres	Primera infancia	n	0	22	0	3	4	1	2	56	88
		%	0,0	25,0	0,0	3,4	4,5	1,1	2,3	63,6	100
	Infancia	n	0	5	0	2	2	4	0	37	50
		%	0,0	10,0	0,0	4,0	4,0	8,0	0,0	74,0	100
	Adolescencia	n	58	11	14	1	2	2	1	34	123
		%	47,2	8,9	11,4	0,8	1,6	1,6	0,8	27,6	100
	Juventud	n	20	3	20	1	0	0	1	45	90
		%	22,2	3,3	22,2	1,1	0,0	0,0	1,1	50,0	100
	Adultez	n	11	0	5	0	0	0	0	23	39
		%	28,2	0,0	12,8	0,0	0,0	0,0	0,0	59,0	100
	Vejez	n	0	1	0	0	0	0	0	3	4
		%	0,0	25,0	0,0	0,0	0,0	0,0	0,0	75,0	100
	Total	n	89	42	44	7	8	7	4	193	394
		%	22,6	10,7	11,2	1,8	2,0	1,8	1,0	49,0	100
Total	Primera infancia	n	0	30	0	5	7	2	2	75	121
		%	0,0	24,8	0,0	4,1	5,8	1,7	1,7	62,0	10%
	Infancia	n	0	7	0	6	2	4	1	56	76
		%	0,0	9,2	0,0	7,9	2,6	5,3	1,3	73,7	100
	Adolescencia	n	59	12	15	1	2	2	1	39	131
		%	45,0	9,2	11,5	0,8	1,5	1,5	0,8	29,8	100
	Juventud	n	21	3	21	1	0	0	1	50	97
		%	21,6	3,1	21,6	1,0	0,0	0,0	1,0	51,5	100
	Adultez	n	11	0	6	0	0	0	0	26	43
		%	25,6	0,0	14,0	0,0	0,0	0,0	0,0	60,5	100
	Vejez	n	0	1	0	0	0	0	0	4	5
		%	0,0	20,0	0,0	0,0	0,0	0,0	0,0	80,0	100
	Total	n	91	53	47	13	11	8	5	245	473
		%	19,2	11,2	9,9	2,7	2,3	1,7	1,1	51,8	100

Fuente: datos del programa de Vigilancia Epidemiológica de Envigado

En el caso de las mujeres, el padre fue el principal victimario durante la primera infancia (25 %) y la infancia (10 %). Cabe señalar que, durante la adolescencia, las mujeres fueron agredidas por los diversos tipos de parientes, aunque principalmente por sus parejas (47,2 %); son estas y las exparejas los parientes que ejercen el mayor porcentaje de violencia sexual contra las mujeres durante la juventud y la adultez (cuadro 3).

Por otra parte, de los 320 casos en los que el agresor no tenía ninguna relación familiar con la víctima, el 51,3 % (164) correspondió a personas desconocidas, el 16,9 %, a conocidos, el 15,0 %, a un amigo, el 7,2 %, a vecinos, el 5,6 %, a un compañero de estudio, el 1,6 %, a un compañero de trabajo, el 1,3 %, al jefe, el 0,6 %, a un profesor, el 0,3 %, a un servidor público y, en el 0,2 % de los casos, no se registró esta información.

Cabe señalar que, en las fichas de notificación de violencia sexual, se encontró información referente a las prácticas de coacción contra las víctimas: en 113 (14,0 %) casos, las víctimas refirieron que sí hubo consumo de alcohol y, en 46 (5,7%), consumo de drogas.

### Tasas poblacionales de violencia sexual

Como ya se señaló anteriormente, en el 74,8 % (604) de los registros se reportó como municipio de residencia Envigado, cantidad utilizada para calcular las tasas poblacionales. En la figura 1 se presenta la tasa general de violencias sexuales por cada 100.000 habitantes en Envigado. Se encontró una tendencia al aumento en el periodo de estudio, con una tasa de 40,5 casos por cada 100.000 habitantes en el 2020.

**Fuente: información:**
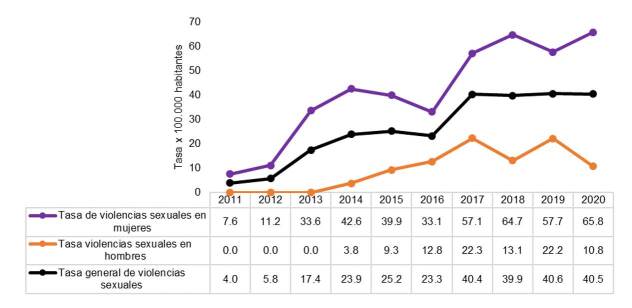
del programa de Vigilancia Epidemiológica de la Secretaría de Salud de Envigado a partir de los datos poblacionales del DANE

Figura 1. Tasa general y específica según sexo de la violencia sexual por cada 100.000 habitantes residentes en Envigado entre el 2011 y el 2020

Al comparar las tasas de violencia sexual según el sexo, se aprecia que fueron superiores en las mujeres que en los hombres (figura 1) e, incluso, superaron la tasa general municipal. En las mujeres, la tasa específica tuvo una tendencia al aumento, siendo la del 2020 la de mayor magnitud: 65,8 casos por cada 100.000 mujeres residentes en Envigado.

**Figura 2:**
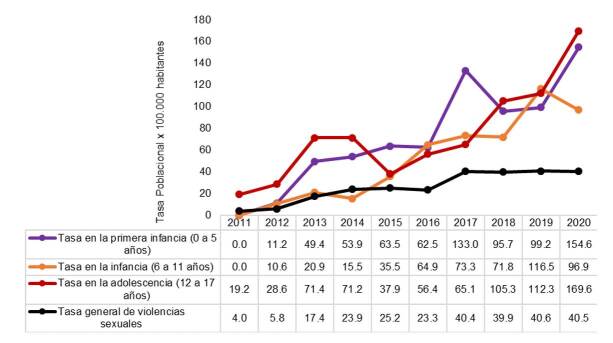
Tasa específica de violencia sexual en la primera infancia, la infancia y la adolescencia por cada 100.000 habitantes residentes en Envigado entre el 2011 y el 2020

En la figura 2 se presentan las tasas poblacionales según la etapa en el curso de vida (primera infancia: 0 a 5 años; infancia: 6 a 11 años, y adolescencia: 12 a 17 años), y se aprecia una tendencia al aumento en todas, especialmente a partir del 2015, cuando superaron la tasa municipal. En la primera infancia, la tasa de violencia sexual más alta se presentó en el 2020, con 154 casos por cada 100.000 niños y niñas entre los 0 y los 5 años. Una situación similar se presentó ese año en la adolescencia, con una tasa de 169 casos por cada 100.000 adolescentes entre 12 y 17 años de edad.

Fuente: información del programa de Vigilancia Epidemiológica de la Secretaría de Salud de Envigado a partir de los datos poblacionales del DANE

**Figura 3:**
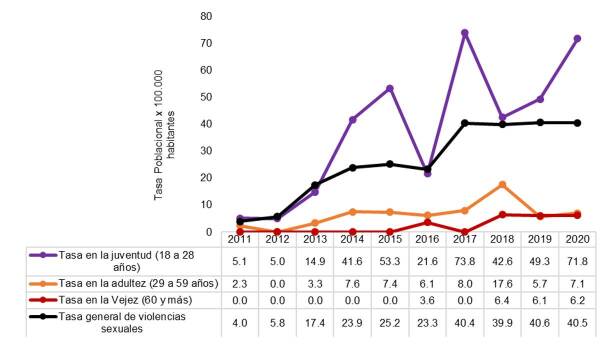
Tasa específica de violencia sexual en la juventud, la adultez y la vejez por cada 100.000 habitantes residentes en Envigado entre el 2011 y el 2020

En las etapas de juventud (18 a 28 años), adultez (29 a 59 años) y vejez (60 y más años) (figura 3), las tasas tuvieron una tendencia estable y de baja magnitud, sobre todo en la adultez y en la vejez; en tanto que, en la juventud, superaron las del nivel municipal, alcanzando una tasa de 73 casos por cada 100.000 jóvenes entre los 18 y los 28 años en el 2017.

Fuente: información del programa de Vigilancia Epidemiológica de la Secretaría de Salud de Envigado a partir de los datos poblacionales del DANE

## Discusión

La notificación de la violencia sexual por parte de los servicios de salud de Envigado aumentó en el periodo de 2011 a 2020. El fenómeno afectó a toda la población, sin distinción de condición social, económica, demográfica o cultural. Las mujeres, en la adolescencia y la juventud, constituyeron la población más afectada, siendo el principal agresor la pareja o la expareja.

La prevalencia de la violencia contra las mujeres y las niñas en la infancia y en la primera infancia, da cuenta de la existencia de patrones culturales que conciben sus cuerpos como objetos sexuales, lo que es propio de culturas machistas, patriarcales y patrilineales ^29^. La sistematicidad y la persistencia en el tiempo de la violencia contra las mujeres dentro de las familias, influyen en su normalización y reproducción en los hijos e hijas de las mujeres víctimas ^12^. En el presente estudio, en estos casos los perpetradores fueron principalmente los padres y los tíos. La literatura especializada reconoce la complejidad de este fenómeno y el impacto que tiene para los niños y las niñas en los aspectos emocional, social, conductual y físico a corto y a largo plazo ^30^^,^^31^. Su detección y manejo implican comprender las barreras que les impiden a los niños y las niñas buscar ayuda.

En un trabajo con supervivientes de violencia sexual durante la infancia, Collin-Vézina, *et al*., señalan tres tipos de barreras: las propias, que incluyen el sentimiento de culpa, los mecanismos o capacidades que se tienen para la autoprotección y el estado de desarrollo biológico y social en el momento del abuso; las relacionales, como la violencia y la disfunción familiar, los roles de poder, el impacto que genera dar a conocer la situación y redes sociales débiles, y las del mundo social, como el estigma, los tabúes sobre la sexualidad, los limitados servicios de atención y el periodo o contexto histórico en el que se produce la situación ^32^.

En estos casos de violencia sexual contra la niñez, también pueden confluir las condiciones de precariedad social y económica de las familias; además, la disfuncionalidad familiar obliga a padres y madres a dejar a los niños y las niñas al cuidado de familiares o personas externas. Para Gonzáles, *et al.*, las condiciones de inestabilidad y ausencia de referentes paternos y, especialmente, el materno, influyen en una limitada vigilancia de los niños y niñas y generan las condiciones que las personas agresoras aprovechan para cometer los abusos sexuales ^33^.

Se apreció un aumento de las tasas poblacionales de violencia sexual en el 2020, año del confinamiento poblacional por la pandemia de la Covid-19, sobre todo en la primera infancia, la adolescencia, la juventud y las mujeres, lo que en parte se explica por la convivencia con el agresor durante dicho periodo. Tales circunstancias pueden exacerbar el trauma y generar crisis de salud mental debido a la recordación de las situaciones traumáticas previas ^34^^-^^36^.

Al comparar estos resultados con los de otro estudio en el departamento de Antioquia, también se constató que las mujeres son las víctimas más frecuentes, así como los adolescentes y los jóvenes. En su estudio sobre la violencia sexual en Antioquia en el marco del conflicto armado, Jiménez, *et al*., encontraron que entre el 2008 y el 2018 se reportaron 4.577 casos de violencia sexual ,y que 49,7 % de ellos fueron en mujeres jóvenes entre los 14 y los 28 años de edad ^37^. En este sentido, el Observatorio de Memoria y Conflicto (OMC) reportó que entre 1959 y el 2020 se registraron 15.760 víctimas de violencia sexual en todo el país, y que el 30,8 % de ellas habían sido niñas y mujeres adolescentes entre los 14 y los 17 años ^11^.

Las mujeres jóvenes y adolescentes suelen ser abusadas por sus parejas y exparejas, lo que da cuenta de noviazgos y relaciones de pareja disfuncionales, dolorosas y violentas, que involucran prácticas de control y manipulación. Ello se explicaría por los procesos de normalización de las vivencias de violencia en sus hogares, los cuales se reproducen de generación en generación e impiden el reconocimiento de la mujer como víctima de violencia sexual y la denuncia del suceso. Además, es importante considerar esta situación porque el noviazgo es uno de los momentos en los que puede iniciarse la dominación y la sumisión de género en las relaciones de pareja ^38^.

Por otro lado, el consumo de alcohol y de drogas es utilizado por los victimarios para coaccionar a las víctimas y justificar los actos de violencia sexual contra ellas, aprovechando la posible pérdida del dominio sobre sus actos y adjudicándoles la responsabilidad por el hecho ^39^. Esto coincide con los hallazgos de otros estudios en otras regiones de Colombia y en países latinoamericanos como Costa Rica y Brasil, donde se ha reportado el consumo de estas sustancias en los casos de violencia sexual ^16^^,^^40^^,^^41^.

Por otra parte, se apreció que, con vínculo familiar o sin él, los hombres en la adolescencia, la juventud y la adultez han sido los principales victimarios en los casos de violencia sexual notificados por los servicios de salud de Envigado. Ello se explicaría a partir de procesos culturales y personales, incluidos la vivencia de experiencias violentas, el abuso durante la infancia y una historia de violencia familiar con prácticas de crianza diferenciadas para hombres y mujeres, que le adjudican a estas los roles de cuidado y atención de otros, y a ellos, el ejercicio de la fuerza física y el poder económico, así como los trastornos psicopatológicos y el consumo de drogas ^42^^,^^43^.

Los casos de violencia sexual en los diferentes grupos étnicos del municipio de Envigado dan cuenta de la existencia de la problemática en estas poblacionales; además, considerando el reducido número de habitantes de grupos étnicos en el municipio (1.507 personas en el 2020, es decir, 0,65 % de la población total), el fenómeno tiene una gran magnitud poblacional, especialmente en la población de los indígenas y los gitanos, que son minoritarias en el municipio ^44^. En este sentido, existe evidencia científica que da cuenta de la magnitud del problema en grupos indígenas de países del continente americano ^45^^-^^47^. En el caso de la población indígena, cabe mencionar las conclusiones de Valdés, *et al*.:

“[…] las mujeres indígenas de las Américas se enfrentan a una triple desventaja, debido a su origen étnico, el género y la segregación; y estas condiciones contribuyen a su mayor vulnerabilidad a la violencia de género y a la discriminación social […]” ^47^.

El presente estudio tiene algunas limitaciones. En primer lugar, la fuente de información secundaria impide el control de la calidad de los registros; también, el constante cambio en las variables de medición de la ficha 875 de vigilancia de la violencia de género e intrafamiliar, podría haber influido en el porcentaje de las variables sin datos; asimismo, cabe reiterar que, en el estudio, se da cuenta de los casos reportados por los servicios de salud y, por ende, puede haber subregistro, pues en muchas situaciones las víctimas no denuncian debido a la coacción que ejercen sus agresores, el desconocimiento de los protocolos de atención, la desconfianza frente a los servicios de salud y la percepción de impunidad y escasa resolución de los casos en Colombia ^48^.

Por otro lado, el aumento de los reportes de violencia sexual a lo largo del tiempo reflejaría, en primer lugar, una mayor capacidad de agencia por parte de la población víctima en la búsqueda de atención en los servicios de salud y en la defensa de sus derechos sexuales, y por otro lado, un mayor cumplimiento por parte de los profesionales de la salud de la Resolución 459 de 2012 del Ministerio de Salud y Protección Social en lo concerniente al reporte de los casos al sistema de vigilancia en salud pública de Envigado ^49^^,^^50^. No obstante, se recomienda a la autoridad sanitaria desarrollar procesos de mejoramiento continuo para que los profesionales de la salud diligencien correctamente todos los campos de las fichas de reporte de este evento, lo que permitirá el desarrollo de políticas públicas respaldadas por la evidencia científica.

En conclusión, la violencia sexual en Envigado constituye un problema de salud pública que atenta contra los derechos humanos de quienes la padecen, especialmente las mujeres, los niños y niñas y la juventud. Esta se produce y se reproduce en el marco de relaciones mediadas por el género, la clase social, la etnia y la generación ^10^^,^^51^, situación que demanda el fortalecimiento de las políticas sociales del municipio contra las inequidades que se expresan en las desigualdades de género y en la respuesta de los servicios de salud para la atención integral de las víctimas.

En este contexto, el desarrollo de programas de tamizaje poblacional y de atención especializada e integral de las víctimas de violencia sexual, es urgente para evitar las consecuencias en la vida de las víctimas, y los costos sociales y económicos que ello acarrea a la administración de Envigado ^52^. Además, se requieren procesos colectivos de transformación cultural de las representaciones sociales de minoridad que se tiene de las mujeres

y la infancia, para lo cual debe haber voluntad política, y el compromiso de implementar la política pública municipal de infancia y adolescencia en coherencia con el bloque de constitucionalidad ^53^^-^^56^.
